# Apparent survival of the salamander *Salamandra salamandra *is low because of high migratory activity

**DOI:** 10.1186/1742-9994-4-19

**Published:** 2007-09-06

**Authors:** Benedikt R Schmidt, Michael Schaub, Sebastian Steinfartz

**Affiliations:** 1 Zoologisches Institut, Universität Zürich, Winterthurerstrasse 190, 8057 Zürich, Switzerland; 2 KARCH, Passage Maximilien-de-Meuron 6, 2000 Neuchâtel, Switzerland; 3 Conservation Biology, Zoologisches Institut, Universität Bern, Baltzerstrasse 6, 3012 Bern, Switzerland; 4 Schweizerische Vogelwarte, 6204 Sempach, Switzerland; 5 University of Bielefeld, Department of Animal Behavior, Morgenbreede 45, 33615 Bielefeld, Germany

## Abstract

**Background:**

Understanding the demographic processes underlying population dynamics is a central theme in ecology. Populations decline if losses from the population (i.e., mortality and emigration) exceed gains (i.e., recruitment and immigration). Amphibians are thought to exhibit little movement even though local populations often fluctuate dramatically and are likely to go exinct if there is no rescue effect through immigration from nearby populations. Terrestrial salamanders are generally portrayed as amphibians with low migratory activity. Our study uses demographic analysis as a key to unravel whether emigration or mortality is the main cause of "losses" from the population. In particular, we use the analysis to challenge the common belief that terrestrial salamanders show low migratory activity.

**Results:**

The mark-recapture analysis of adult salamanders showed that monthly survival was high (> 90%) without a seasonal pattern. These estimates, however, translate into rather low rates of local annual survival of only ~40% and suggest that emigration was important. The estimated probability of emigration was 49%.

**Conclusion:**

Our analysis shows that terrestrial salamanders exhibit more migratory activity than commonly thought. This may be due either because the spatial extent of salamander populations is underestimated or because there is a substantial exchange of individuals between populations. Our current results are in line with several other studies that suggest high migratory activity in amphibians. In particular, many amphibian populations may be characterized by high proportions of transients and/or floaters.

## Background

Local population dynamics are a function of survival and reproductive rates within a focal area and rates of dispersal into and out of that area. The spatial extent and the degree of isolation of a local population have an impact on local population dynamics and hence these factors need to be considered in studies about local population dynamics [1-3]. Only if we understand the patterns in vital rates – birth, death, emigration and immigration – and the processes that cause these patterns, then can we understand the ecology and evolutionary dynamics of a species.

The four main drivers of population change can be conveniently summarized as gains (birth and immigration) and losses (mortality and emigration). Theory for species with complex life cycles, such as amphibians, predicts and empirical studies of amphibian populations confirm that a change in "losses" has a stronger impact on amphibian population growth rate than a same change in the "gains" [4,5]. We use data from a two-year mark-recapture study of adult fire salamanders (*Salamandra salamandra*) to address two questions on how the two types of "losses", mortality and emigration, affect amphibian population dynamics. First, we ask when losses occur, because the timing and type of losses may affect population dynamics [6]. Second, terrestrial salamanders like *Salamandra salamandra *are generally considered to display strong site fidelity to small home ranges and move little during an individual's adult life [7-9]. This classic view, however, is beginning to change [10-14]. Therefore, we ask what proportion of salamanders emigrate from the study area and whether the emigration component of losses from the population could have a large influence on population change.

Our first question regarding losses is whether survival varies seasonally with a difference in adult survival between summer – when individuals are active – and winter, when individuals are inactive. Seasonal variation provides information when most mortality occurs during the annual cycle and therefore indicates at which time mechanisms of population regulation operate. The distinction between winter and summer mortality is crucial as they are likely influenced by different factors. Winter survival is almost certainly determined by extrinsic environmental factors such as weather conditions [15]. Summer survival, in contrast, could be more affected by either intrinsic factors that may depend on population density or behaviour. Accordingly, summer and winter survival may be under hard or soft selection, respectively, and this may profoundly affect population dynamics [6].

The second issue we address is migration. Migration of amphibians has received relatively little attention in the past, but it seems that it is probably far more important than hitherto thought for the dynamics of populations ranging from patchy to metapopulations [10]. At small spatial scales, movement of individuals determines the spatial extent of a population and patterns of genetic differentiation within and among demes. We are only beginning to understand that amphibian populations have a much larger spatial extent than it is commonly thought [10-14]. At larger spatial scale, migration likely affects the persistence of populations and metapopulations. Amphibian populations are well-known for large fluctuations in abundance [16]. With such large fluctuations, individual populations have a high risk of extinction [17,18]. Thus, migration between populations is essential to prevent local extinction (i.e., the rescue effect [19]). *Salamandra salamandra *is well suited to address these questions because until recently it was considered as a text book example for strong site fidelity and small home ranges [7-9].

Here, we report estimates of survival of adult salamanders that are remarkably low in comparison with estimates from previous studies. We then show that low apparent survival is likely to be the consequence of high emigration rates, thus confirming the emerging view that amphibians are far more vagile than commonly assumed.

## Results

We captured 86 individuals of which 41 individuals had been recaptured at least once. A summary of the data is provided in Table 1. The general model with time-dependent apparent survival and recapture probabilities (φ_t_, *p*_t_) fitted the data well (goodness-of-fit test with U-CARE [19]: χ^2 ^= 1.574, df = 4, *P *= 0.813). Thus, there is no evidence for heterogeneity in detection probabilities (which might have been caused by salamanders that inhabit home ranges close to the edge of the study area and that may temporarily leave the study area).

**Table 1:** Summary of the capture-recapture data of 86 individual fire salamanders from Ellhauser Forest (Germany) collected between 2001 and 2003 (m-array format, [47]).

		Number of recaptures			
		
Occasion	Releases	Sept. 2001	May 2002	Sept. 2002	May 2003
May 2001	80	17	17	2	1
Sept. 2001	17		11	0	0
May 2002	34			12	5
Sept. 2002	14				3

For each capture occasion the number of released individuals and the number of recaptured individuals of each release cohort per occasion is shown.

**Figure 1:**
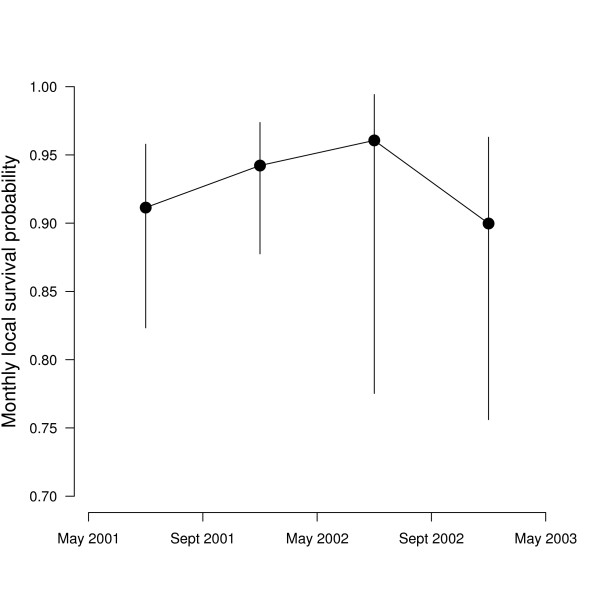
**Model averaged monthly apparent survival probability of *Salamandra salamandra *from Ellhauser Forest (Germany)**. The vertical lines show the limits of the unconditional 95% confidence intervals.

Due to the relatively small sample size and the short duration of the study, there was model selection uncertainty, i.e., several models explained well the information in the data (Table 2). Models with fewer parameters had generally higher support from the data than more complex models. Still, the poor fit of model (φ_._, *p*_._) indicates that there was structure in the data. Instead of focusing on a single best model for inference, we computed model averaged parameter estimates [21]. Model averaged monthly survival probabilities were greater than 0.9 and increased initially but then dropped during the last winter (Figure 1).

**Table 2:** Modelling monthly apparent survival (φ) and recapture probabilities (*p*) of fire salamanders from Ellhauser Forest (Germany).

Model	Deviance	K	ΔAICc	*w*_i_
φ_t_, *p*_s_	14.93	6	0.00	0.33
φ_._, *p*_t_	17.51	5	0.40	0.27
φ_._, *p*_s_	23.13	3	1.76	0.13
φ_t_, *p*_t_	14.92	7	2.21	0.11
φ_s_, *p*_t_	17.44	6	2.52	0.09
φ_s_, *p*_s_	23.10	4	3.85	0.05
φ_s_, *p*_._	30.13	3	8.76	0.00
φ_t_, *p*_._	26.67	5	9.56	0.00
φ_._, *p*_._	36.60	2	13.14	0.00

The model subscript t refers to time dependence (i.e., different in each year and season), s denotes a seasonal effect (i.e., different survival during summer and winter months), and a dot (.) denotes constancy. Table entries are deviance (Deviance) of each model, the number of estimated parameters (*K*), the difference of the AICc value of the current model and of the best model (ΔAICc), and the AICc weight (*w*_i_).

We calculated the annual survival probability as the product of the monthly survival probabilities. We assumed that the summer period lasts 5 months (May to September) and the winter period 7 months (October to April), thus annual apparent survival probability resulted as φ_summer_^5^*φ_winter_^7^. The corresponding standard error was calculated by applying the delta method [22]. The annual apparent survival probabilities were 0.41 (SE: 0.16) and 0.39 (SE: 0.28) for the two years, respectively.

Our estimates of apparent survival are confounded with permanent emigration. That is, apparent survival is equal to true survival multiplied by (1 – probability of emigration) [23]. We can thus calculate the probability of emigration as 1 – (apparent survival/true survival). Based on the survival estimates for *Salamandra salamandra *in a very similar habitat presented in [5], we assumed that true survival would be around 0.8. This yielded an estimate of the annual probability of emigration of 0.49.

**Figure 2:**
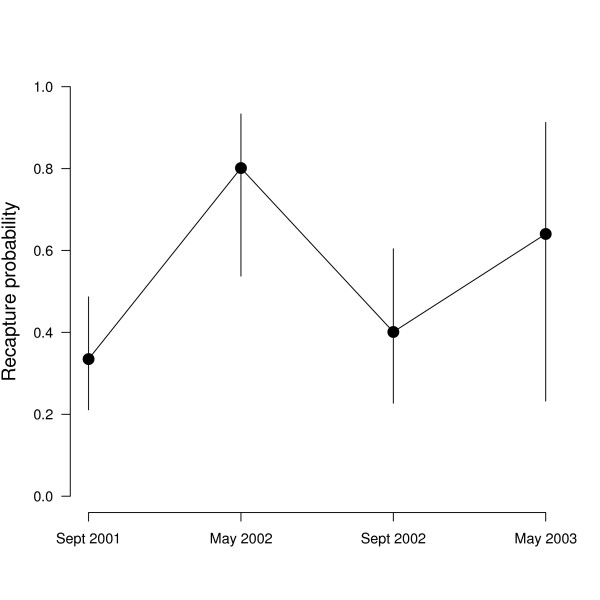
**Model averaged recapture probability of *Salamandra salamandra *from Ellhauser Forest (Germany)**. The vertical lines show the limits of the unconditional 95% confidence intervals.

Recapture probabilities showed a clear seasonal pattern. They were low during autumn and high during the spring capture sessions (Fig. 2). Such a pattern may lead to erroneous conclusions if recapture probabilities are not accounted for in the analysis [24].

## Discussion

The mark-recapture analysis of adult fire salamanders showed that monthly apparent survival varied through time. Estimates of apparent annual survival were remarkably low and suggest that emigration from the study site is strong: about 50% of the salamanders emigrated from the study area. Our results corroborate the emerging view that amphibians are far more mobile than commonly thought [10-14,16,19].

Apparent monthly survival varied from month to month but not seasonally. Reading [15] reported that survival of common toads was a function of winter weather. In salamanders, however, there is apparently no season during which mortality is elevated [2]. It may be that different factors affect survival in different months but their net effect on survival appears to be the same. Hence, our study does not reveal whether extrinsic factors, such as harshness of winters, or behaviourally- or density-mediated factors during the activity period primarily affect salamander survival and consequently population dynamics. We believe that further study of seasonal variation in salamanders is warranted because different factors may exert soft or hard selection on salamanders that in turn determine whether populations are regulated locally or at the metapopulation level [6].

Annual apparent survival of the Ellhauser forest salamander population was considerably lower than other estimates [5]. While there is certainly geographic variation in life history traits [25], survival rate of the Ellhauser forest population is half that of another well studied German population of *Salamandra salamandra *[5]. The most plausible explanation for this difference is a relatively high rate of emigration from our study site. The indirect estimate of the probability of emigration showed that about 50% of the salamanders left the study area. This is a large proportion in light of the strong site fidelity and small home ranges that have been reported for this salamander species [7,26,27]. Our analysis suggests that salamanders may be using large home ranges or may have emigrated to neighbouring populations. Indeed, data from the same population show that salamanders in the Ellhauser forest use large home ranges (average 494 m^2^) and moved on average 52 m and 117 m per season in 2001 and 2002, respectively. Average distance between successive recaptures was 64 m and ranged from 4 to 319 m. Thus, many are likely to have left our study area, which had an area of 290'000 m^2 ^(equivalent to a circle with a radius of 300 m [28]). Given the design of our study, we must currently treat migration as a yes/no event where we categorize individuals as "stayers" and "movers". The necessary next step will have to be the better characterization of the movement distribution of salamanders [2].

Further evidence corroborates the hypothesis of high migratory activity in salamander populations. The salamander population studied by [5] showed a large proportion of transients and provided evidence for temporary emigration. Transients are animals that are encountered only once and then leave the study area [29]. Temporary emigrants are salamanders that leave the study area for a while, then return. Because temporary emigrants appear as permanent emigrants in a short-term study such as ours, we cannot discriminate between temporary or permanent emigration. Temporary emigration indeed could result from home ranges that are larger than the study area. Nevertheless, our analysis and the spatial analysis of [28] clearly provide evidence for the fact that terrestrial salamanders can be highly vagile. Further evidence for high migratory activity of *S. salamandra *comes also from population genetic analyses which showed genetic uniformity of salamander populations in an area (the Eifel forest) of more than 225 km^2 ^[14].

These data on migratory activity contrast strongly with the common view of amphibians, and salamanders in particular, being characterized by low vagility [10,30-34], but supports the alternative view that amphibians show high vagility [10]. Closer inspection of mark-recapture studies reveals further evidence that many amphibians appear to migrate substantially. Several studies found evidence for transients [11,15,35-37]. Transients are animals that are encountered only once and then leave the study area [28]. In mark-recapture analyses, transients are detected as violations of assumptions of the traditional Cormack-Jolly-Seber model. Although alternative explanations for the violations of assumptions are possible and favoured by some authors (e.g., effects of marking on survival [37]), nomadic animals on the move that show high vagility and little site fidelity seem a likely explanation, especially if alternative explanations do not apply (e.g., where marking is non-invasive [5]). Proportions of transients can be high. For example, [35] estimated the proportion of transients in the toad *Bufo bufo *as 0.43 (CI 0.27–0.59) in one year and 0.54 (CI 0.32–0.75) in another year, whereas [11] report an average proportion of 0.35. Interestingly, there may be variation among populations in their ability to migrate or show "nomadic" behaviour. [5] only found evidence for transients in one of the two populations that they studied. Transients would perhaps be largely equivalent to non-territorial floaters that have been reported for many vertebrate groups, including amphibians [38]. Floaters may be important for population dynamics and conservation [39-41].

The accumulated evidence suggests that amphibians are more mobile than commonly thought. We still need to fully understand the behavioural mechanisms that underlie individual movement patterns as there are several explanations for the observed patterns. Both high rates of emigration and transients may be either due to inappropriate definition of the spatial extent of amphibian populations or may reflect migration between populations. The delineation of the study area clearly affects any inference regarding dispersal and needs to be done with great care [3]. In any case, movement behaviour may be a key to understanding the temporal and spatial dynamics of amphibian populations [10,12,19]. Rescue effects caused by high numbers of migrants could ensure the persistence of amphibian populations despite the strong fluctuations that characterize amphibian populations [16].

Finally, a better understanding of amphibian movement behaviour is also important for a better understanding of the evolutionary dynamics of amphibians such as patterns of genetic differentiation between amphibian populations. Most of these patterns are interpreted as the result of passive differentiation mechanisms [42]. However, patterns of genetic differentiation should not only be explained by limited dispersal ability of amphibians. The alternative view is that amphibian populations may show high rates of divergence that are adaptive rather than the result of passive processes [43,44].

## Conclusion

Migration to and from populations affects the local dynamics of animal populations. We focus on the demographics of amphibians, a highly threatened group of vertebrates that are commonly thought to show little movement. The analysis of mark-recapture data from a population of salamanders gave remarkably low estimates of apparent survival. These estimates suggest that the probability of emigration was high. Such a high rate of emigration implies that either the spatial extent of salamander populations is greatly underestimated or else migration rates between populations are indeed very high. Both possible interpretations suggest that salamanders are much more mobile than commonly thought. The evidence for transients in mark-recapture studies of a large number of amphibian populations provides indirect evidence for a substantial proportion of transients, i.e., animals on the move. Thus, the results support and extend the emerging new view of amphibians as highly vagile animals. Such a new view means that we would have to reformulate our hypotheses as to how amphibian populations function and suggest that conservationists should strive to maintain migratory activity within and among amphibian populations because migration may be the key determinant of population viability.

## Methods

### The species and study site

**Figure 3:**
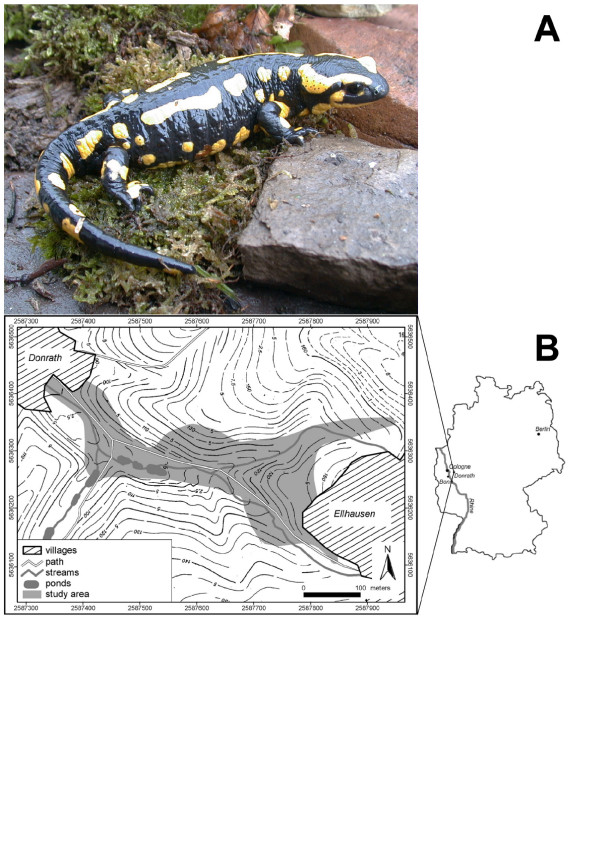
A. Adult fire salamander (*Salamandra salamandra*) as typically found in the study area. B. Sketch of the study area.

*Salamandra salamandra *is a terrestrial salamander (Figure 3A) that inhabits old broadleaf forest with many small streams. During spring, females migrate to small streams where they give birth to larvae. The streams serve as the habitat for the larvae whereas juveniles and adults are terrestrial. Mating also occurs on land. Salamanders normally start to reproduce after 3–5 years and are long-lived [5,9].

Our study site is part of the Ellhauser Forest located in western Germany 25 km south-east of Cologne near Donrath within the Bergisches Land (see figure 3B). The forest in which salamanders were found is mainly composed of beech (*Fagus sylvatica*) and oak (*Quercus robur*) and represents a typical habitat of *S. salamandra*. Its average altitude is around 200 m above sea level and the annual precipitation is around 1100 mm/m^2^.

### Data collection and mark-recapture analysis

Adult salamanders larger than 12 cm in total length were fitted with a Euro-I. D. bio-glas transponder (weight 0.09 g, length 12 mm, diameter 2.2 mm). For details of the implantation process of PIT tags see [28]. Altogether 86 individuals (59 females, 23 males and 4 individuals of unknown sex) were fitted with PIT tags and released at the site of initial capture.

Field visits were conducted to locate marked individuals. These consisted of repeated intensive searches using torches by 2–3 persons for 5–6 hours from April 2001 until May 2003 in a fixed sub-area of the Ellhauser forest (corresponding to 0.290 km^2^). Each salamander found was scanned with a LID-500 hand scanner and if a transponder was detected, both the corresponding transponder code and the coordinates (Gauss-Krueger) of the recapture site were recorded using a differential Global Positioning System (GPS) with an accuracy of three meters. The captures were carried out almost continuously from April to beginning of July, and a second time from September until late October (beginning of hibernation). We pooled all observations from different years conducted in the months of March to July to the occasion "spring" and all captures conducted in the months of September and October to the occasion "autumn". This pooling procedure does not cause bias in survival estimates [45]. The mean capture dates in these five occasions were 7.5.2001, 14.9.2001, 24.4.2002, 28.9.2002 and 2.5.2003. We defined apparent survival from May to September as "summer survival" and apparent survival from September to May as "winter survival".

We analysed the capture-recapture data with Cormack-Jolly-Seber models [46], which allow separate estimates of apparent survival and recapture probabilities. The apparent survival probability (φ_i_) is the probability that an individual that is in the population at time *i *is still alive and in the population at time *i*+1. This implies that true mortality and permanent emigration are confounded. The recapture probability (*p*_i_) is defined as the probability of sighting a marked individual that is alive and in the population at time *i*. The estimation of these parameters requires several assumptions to be met, which can be tested with goodness-of-fit tests [35,47,48]. Once a global model has been found to fit the data adequately, nested models can be fitted and their support from the data assessed using Akaike's Information Criterion (AIC_c _[21]).

We used program MARK [49] to estimate apparent survival and recapture probabilities. We accounted for the unequal time intervals between capture occasions and estimated monthly survival rates.

We fitted a small set of candidate models to the data. Survival was modelled as either constant through time, varying through time (but with no particular pattern) or with a seasonal effect (i.e., summer and winter survival different). The probability of recapture was modelled in the same way: constant, time-varying, or seasonal.

## Competing interests

The author(s) declare that they have no competing interests.

## Authors' contributions

SS initiated this cooperation as a part of his ongoing research on mechanisms of speciation in terrestrial salamanders, provided the capture recapture data as well as manuscript preparation. MS and BRS designed the analysis that was performed by MS. BRS drafted the manuscript. All authors read and approved the final manuscript.

## References

[B1] Clobert J, Danchin E, Dhondt AA, Nichols JD, eds (2003). Dispersal.

[B2] Lowe WH (2003). Linking dispersal to local population dynamics: a case study using a headwater salamander system. Ecology.

[B3] Schaub M, Ullrich B, Knötzsch G, Albrecht P, Meisser C (2006). Local population dynamics and the impact of scale: a case study on different little owl populations. Oikos.

[B4] Biek R, Funk WC, Maxell BA, Mills LS (2002). What is missing in amphibian decline research: Insights from ecological sensitivity analysis. Cons Biol.

[B5] Schmidt BR, Feldmann R, Schaub M (2005). Demographic processes underlying population growth and decline in *Salamandra salamandra *. Cons Biol.

[B6] Saccheri I, Hanski I (2006). Natural selection and population dynamics. Trends Ecol Evol.

[B7] Joly J (1963). La sédentarité et le retour au gite chez la Salamandra tâchetée. C R Acad Sci.

[B8] Pough FH, Andrews RM, Cadle JE, Crump ML, Savitzky AH, Wells KD, 2 (2001). Herpetology.

[B9] Thiesmeier B, Grossenbacher K, Thiesmeier B, Grossenbacher K (2004). *Salamandra salamandra *(Linnaeus, 1758) – Feuersalamander. Handbuch der Reptilien und Amphibien Europas: Schwanzlurche IIB.

[B10] Smith MA, Green DM (2005). Dispersal and the metapopulation paradigm in amphibian ecology and conservation: are all amphibian populations metapopulations?. Ecography.

[B11] Perret N, Pradel R, Miaud C, Grolet O, Joly P (2003). Transience, dispersal and survival rates in newt patchy populations. J Anim Ecol.

[B12] Petranka JW, Smith CK, Scott AF (2004). Identifying the minimal demographic unit for monitoring pond-breeding amphibians. Ecol Appl.

[B13] Funk WC, Greene AE, Corn PS, Allendorf FW (2005). High dispersal in a frog species suggests that it is vulnerable to habitat fragmentation. Biol Lett.

[B14] Steinfartz S, Weitere M, Tautz D (2007). Tracing the first step to speciation – ecological and genetic differentiation of a salamander population in a small forest. Mol Ecol.

[B15] Reading CJ (2007). Linking global warming to amphibian declines through its effects on female body condition and survivorship. Oecologia.

[B16] Green DM (2003). The ecology of extinction: population fluctuation and decline in amphibians. Biol Cons.

[B17] Schoener TW, Spiller DA (1992). Is extinction rate related to temporal variability in population size? An empirical answer for orb spiders. Amer Nat.

[B18] Inchausti P, Halley J (2003). On the relation between temporal variability and persistence time in animal populations. J Anim Ecol.

[B19] Green DM, Lannoo M (2005). Biology of amphibian declines. Amphibian declines: The conservation status of United States species.

[B20] Choquet R, Reboulet AM, Lebreton JD, Gimenez O, Pradel R (2005). U-CARE 2.2 User's Manual. CEFE, Montpellier, CEFE.

[B21] Burnham KP, Anderson DR (2002). Model selection and multimodel inference: a practical information-theoretic approach.

[B22] Seber GAF (1982). The estimation of animal abundance and related parameters.

[B23] Burnham KP, Lebreton JD and North PM (1993). A theory for combined analysis of ring recovery and recapture data. The study of bird population dynamics using marked individuals.

[B24] Schmidt BR (2004). Declining amphibian populations: The pitfalls of count data in the study of diversity, distributions, dynamics, and demography. Herpetol J.

[B25] Berven KA, Gill DE (1983). Interpreting geographic variation in life history traits. Amer Zool.

[B26] Denoël M (1996). Phénologie et domaine vital de la *Salamandra salamandra terrestris *(Amphibia, Caudata) dans un bois du Pays de Herve (Belgique). Cahiers Ethol.

[B27] Rebelo R, Leclair MH (2003). Site tenacity in the terrestrial salamandrid, *Salamandra salamandra *. J Herpetol.

[B28] Schulte U, Küsters D, Steinfartz S (2007). A PIT tag based analysis of annual movement patterns of adult fire salamanders (*Salamandra salamandra*) in a Middle European habitat. Amphibia-Reptilia.

[B29] Pradel R, Hines JE, Lebreton JD, Nichols JD (1997). Capture-recapture survival models taking account of transients. Biometrics.

[B30] Gill DE (1978). Effective population size and interdemic migration rates in a metapopulation of the red-spotted newt, *Notophthalmus viridescens *(Rafinesque). Evolution.

[B31] Sinsch U (1990). Migration and orientation in anuran amphibians. Ethol Ecol Evol.

[B32] Blaustein AR, Wake DB, Sousa WP (1994). Amphibian declines: Judging stability, persistence and susceptibility to local and global extinctions. Cons Biol.

[B33] Stebbins RC, Cohen NW (1995). A natural history of amphibians.

[B34] Beebee TJC (2005). Conservation genetics of amphibians. Heredity.

[B35] Schmidt BR, Schaub M, Anholt BR (2002). Why you should use capture-recapture methods when estimating survival and breeding probabilities: on bias, temporary emigration, overdispersion, and common toads. Amphibia-Reptilia.

[B36] Frétey T, Cam E, Le Garff B, Monnat JY (2004). Adult survival and temporary emigration in the common toad. Can J Zool.

[B37] Scherer RD, Muths E, Noon BR, Corn PS (2005). An evaluation of weather and disease as causes of decline in two populations of boreal toads. Ecol Appl.

[B38] Mathis A (1991). Territories of male and female terrestrial salamanders: costs, benefits, and intersexual spatial associations. Oecologia.

[B39] Durell SEA le V dit, Clarke RT (2004). The buffer effect of non-breeding birds and the timing of farmland bird declines. Biol Cons.

[B40] Lopez-Sepulcre A, Kokko H (2005). Territorial defense, territory size, and population regulation. Amer Nat.

[B41] Penteriani V, Otalora F, Ferrer M (2006). Floater dynamics can explain positive patterns of density-dependent fecundity in animal populations. Amer Nat.

[B42] Larson A, Wake DB, Yanev KP (1984). Measuring gene flow among populations having high-levels of genetic fragmentation. Genetics.

[B43] Skelly DK (2004). Microgeographic countergradient variation in the wood frog, *Rana sylvatica *. Evolution.

[B44] Van Buskirk J, Arioli M (2005). Habitat specialization and adaptive phenotypic divergence of anuran populations. J Evol Biol.

[B45] O'Brien S, Robert B, Tiandry H (2005). Consequences of violating the recapture duration assumption of mark-recapture models: a test using simulated and empirical data from an endangered tortoise population. J Appl Ecol.

[B46] Lebreton JD, Burnham KP, Clobert J, Anderson DR (1992). Modeling survival and testing biological hypothesis using marked animals: a unified approach with case studies. Ecol Monogr.

[B47] Burnham KP, Anderson DR, White GC, Brownie C, Pollock KH (1987). Design and analysis methods for fish survival experiments based on release-recapture. American Fisheries Society Monograph Number 5.

[B48] Schaub M, Gimenez O, Schmidt BR, Pradel R (2004). Estimating survival and non-random temporary emigration in the multistate capture-recapture framework. Ecology.

[B49] White GC, Burnham KP (1999). Program MARK: survival estimation from populations of marked animals. Bird Study.

